# Comparison of the efficacy and safety of super-selective and selective transcatheter arterial embolization in non-variceal gastrointestinal bleeding

**DOI:** 10.3389/fmed.2025.1697511

**Published:** 2025-12-01

**Authors:** Jiawei Zhong, Kang Li, Taiyu Chen, Foqiang Liao, Xu Shu

**Affiliations:** Department of Gastroenterology, Jiangxi Provincial Key Laboratory of Digestive Diseases, Jiangxi Clinical Research Center for Gastroenterology, Digestive Disease Hospital, The First Affiliated Hospital, Jiangxi Medical College, Nanchang University, Nanchang, Jiangxi, China

**Keywords:** acute nonvariceal gastrointestinal bleeding, selective embolization, super-selective embolization, clinical success, rebleeding

## Abstract

**Background:**

Vascular interventional therapy (TAE) is an effective treatment for most abdominal organ hemorrhages, especially for non-variceal gastrointestinal bleeding (NVGIB) unresponsive to endoscopy. While selective and super selective embolization are two key interventional hemostasis methods, research on their application in total gastrointestinal bleeding is limited. This study compares these two techniques to assess their safety in non-variceal gastrointestinal bleeding hemostasis.

**Methods:**

We conducted a retrospective study on patients with NVGIB who received vascular interventional therapy from August 2014 to October 2024, comparing the clinical characteristics and outcomes of patients with selective or super-selective embolization. The primary outcome was clinical success, and secondary outcomes included technical success, rebleeding (overall and within 3 days), transfusion requirements, need for additional therapies, complications, and mortality.

**Results:**

A total of 116 patients with NVGIB who received vascular interventional therapy were included. Among the 88 patients with non-variceal upper gastrointestinal bleeding (NVUGIB), 48 and 40 were treated with super-selective and selective embolization, respectively. Gastroduodenal artery was the most common embolized vessel in both groups. All cases achieved technical success. 85.42% of the super-selective embolization group achieved clinical success, and 70.00% of the selective embolization group achieved clinical success (*p =* 0.080). The rebleeding within 3 days rate of the super-selective embolization group was significantly lower than that in the selective embolization group (8.33% vs. 27.50%, *p* = 0.017). The bleeding related mortality was 6.25% in the super-selective embolization group and 7.50% in the selective embolization group. In the subgroup of 28 patients with lower gastrointestinal bleeding (LGIB), no significant differences in clinical outcomes were observed between the two embolization approaches. However, it is noteworthy that all 5 cases of post-procedural intestinal ischemia occurred in this LGIB subgroup. The embolic material used had a significant impact on early rebleeding and the subsequent need for additional therapy in LGIB (*p* < 0.05), but not in UGIB.

**Conclusion:**

For refractory NVUGIB, super-selective TAE compared to selective TAE reduces early rebleeding. Decision-making should prioritize patient transfusion needs, which was the sole independent predictor of rebleeding. The embolization strategy for LGIB should carefully consider the choice of embolic material and the inherent risk of intestinal ischemia.

## Introduction

Acute nonvariceal gastrointestinal bleeding (NVGIB) is a severe digestive system disease, characterized by rapid changes in condition, difficulty in accurately estimating blood loss, and a tendency to recur. Despite recent advances in endoscopy and pharmacological therapy, NVGIB mortality remains at 3–14% (1, 2). Lower gastrointestinal bleeding mortality is slightly lower, but severe lower gastrointestinal bleeding (LGIB) still has a 5—year mortality rate of up to 13% (3). Transcatheter arterial embolization (TAE) is typically used as a rescue treatment for NVGIB when endoscopic therapy fails (4). Compared with other treatments, TAE is less invasive, more accurate in localization, faster in hemostasis, and has wider applicability (5–7). Recent studies have confirmed the safety and effectiveness of TAE in gastrointestinal hemostasis. For non-variceal upper gastrointestinal bleeding (NVUGIB), TAE has a high technical success rate of 94.9% and a clinical success rate of 73.0% (4).

Selective and super-selective embolization are now the two main TAE methods for hemostasis. Selective embolization involves infusing embolic agents through a catheter into the main trunk or branches of target blood vessels to block local blood flow, offering a wide control range (8). Super-selective embolization, performed in finer vessels and targeting specific lesion areas or branches, is characterized by precise hemostasis (9). Numerous studies have compared these two embolization techniques in vascular interventional fields, especially in abdominal blood vessels such as the hepatic artery, renal artery, ureteral artery, and bladder artery (10–12). Concurrent with ongoing technological advances, both selective and super—selective embolization have demonstrated significant advancement in technical process, embolic agent selection, and indication criteria for NVGIB compared to previous non—selective embolization (6, 13). However, their comparative effectiveness in clinical use remains controversial. A retrospective study demonstrated that in hemodynamically unstable emergency patients, selective embolization has a higher technical success rate due to its low dependence on vascular conditions and equipment (14). Especially when DSA does not clearly identify bleeding sites, selective embolization can “blind—embolize” suspected blood vessels, effectively covering potential bleeding branches (15). Some studies indicate that super—selective embolization, which uses microcatheters (3 Fr) to deliver embolic materials to terminal arteries near bleeding sites, significantly reduces the risk of bowel ischemia compared to selective embolization (16).

Given the limited evidence on the comparative efficacy and safety of superselective and selective embolization in NVGIB patients with endoscopic treatment failure, we designed a retrospective study to evaluate the clinical outcomes of these two techniques in such patient populations.

## Methods

### Study design

Patients diagnosed with NVGIB and treated with abdominal angiography at our hospital from August 2014 to October 2024 were included, all cases were consecutive. All patients presented with clinical symptoms such as hematemesis, melena, or hematochezia, and underwent endoscopic examination or enhanced CT. The final diagnosis was made using digital subtraction angiography (DSA). Exclusion criteria were as follows: (1) Patients with negative angiography or no interventional embolization; (2) Patients with hemorrhage from the biliary tract, pancreas, or malignancy; (3) Patients with incomplete or missing data; the study was approved by our hospital’s ethics committee, and patients’ informed consent was exempted.

### Variables

The baseline information incorporated gender, age, comorbidities (hypertension, diabetes, heart disease, liver cirrhosis, renal failure, abdominal surgical history), lifestyle factors (smoking, alcohol consumption), clinical data (pre-TAE blood pressure), time interval from bleeding to TAE (<6 h or not), Clip use, endoscopic and angiographic findings, blood transfusion details (including units transfused), and pre-TAE laboratory parameters [white blood cell count (WBC), hemoglobin (HB), platelet count (PLT), albumin, Blood urea nitrogen (BUN), creatinine (Cr), International Normalized Ratio (INR), Prothrombin Time (PT)]. The outcome measures comprised rebleeding rates (overall and﹤3 days), additional treatments required for rebleeding (endoscopy, re-embolization, surgery), Postoperative complications (fever, abdominal pain, nausea/vomiting, Intestinal ischemia), length of hospital stay, and mortality. Technical success is defined as the immediate termination of intravascular bleeding through TAE, that is, angiography shows no extravasation. Clinical success is defined as no recurrence of gastrointestinal bleeding within 30 days after TAE (17). Bleeding-related mortality was defined as deaths directly attributable to uncontrollable gastrointestinal bleeding.

### Operating procedures

All TAE procedures were performed by experienced interventional radiologists. Patients were placed in the supine position, given local infiltration anesthesia, and percutaneous femoral puncture was performed using a 5 Fr catheter for angiography of the celiac axis, gastroduodenal artery, left gastric artery, superior and inferior mesenteric arteries. Selective angiography of branches was then done based on bleeding situation. The direct positive angiographic sign was considered as contrast agent extravasation, and aneurysms/pseudoaneurysms, vascular irregularities, vessel cutoffs, arteriovenous/arterioportal shunts, neovascularization, and increased small arterial vessels were regarded as indirect signs of bleeding, indicating embolization. If contrast agent extravasation or indirect bleeding signs were found, selective embolization was performed on the trunk or branch vessels. Otherwise, super—selective angiography was done as close to the bleeding site as possible using a 2.7 Fr microcatheter (Progreat, Terumo, Tokyo, Japan), and super—selective embolization was performed based on the vascular conditions. Some patients underwent prophylactic embolization according to endoscopic findings of the bleeding site and the operator’s experience. The embolization materials included spring coil and gelatin sponge. Post—TAE angiography was performed to confirm occlusion of the bleeding site. If bleeding remained uncontrolled, repeat angiography or surgical referral was done.

### Statistical methods

Quantitative data distribution was determined using the Shapiro—Wilk normality test. Normally distributed variables were presented as mean ± standard deviation (SD), and skewness—distributed ones as median (interquartile range, IQR). Categorical data were presented as numbers and ratios. Student’s t—test or Mann–Whitney U test was used for quantitative data analysis, and χ^2^ test or Fisher’s exact test for categorical data. *p*-value < 0.05 was considered statistically significant. For univariate analysis, variables with *p* < 0.05 were selected; multivariate analysis was subsequently performed using logistic regression. All statistical analyses were conducted using R software (version 4.2.0, R Foundation for Statistical Computing, Vienna, Austria).

## Results

### Baseline characteristics

**Figure 1:**
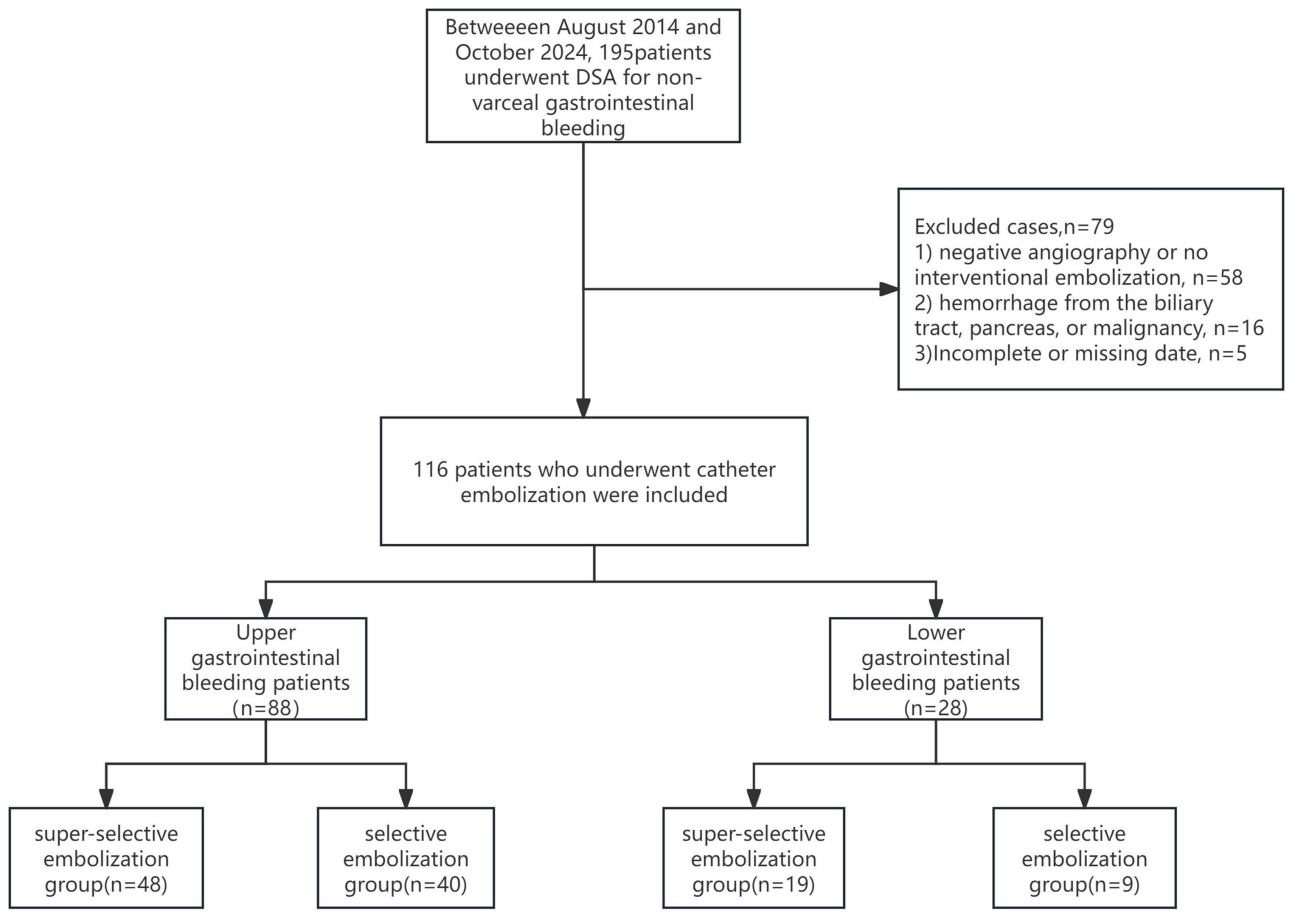
Flow charts of included patients.

Figure 1 shows the flowchart of this study. From August 2014 to October 2024, a total of 116 patients with NVGIB who received vascular interventional therapy were included. As shown in Table 1, Of the 88 patients with NVUGIB, 48 underwent super-selective embolization and 40 received selective embolization. The median (IQR) age of patients in the super-selective embolization group and the selective embolization group was 65.50 (53.75, 73.25) and 59.00 (43.75, 72.50) years, respectively. The proportion of patients with positive results under endoscopy in the super-selective embolization group was 72.92% (35/48), while that in the selective embolization group was 85.00% (34/40). There was no significant difference in the proportion of patients receiving blood transfusion between the two groups. However, the mean blood transfusion units in the selective embolization group were 4.55 ± 5.20, which was significantly higher than that in the super-selective embolization group (*p* = 0.017). There were no significant differences between super-selective embolization group and selective embolization group in terms of gender, alcohol, smoking, laboratory index, time to treatment ≤6 h and etc. (*p >* 0.05). Similarly, Supplementary Table 1 summarizes the baseline characteristics of the 28 patients with lower gastrointestinal bleeding, of whom 19 underwent super-selective embolization and 9 received selective embolization. No statistically significant differences were observed in any variables between the selective and super-selective embolization groups (*p >* 0.05).

**Table 1:** Baseline characteristics of patients with NVUGIB.

Variables	Super-selective embolization group (*N* = 48)	Selective embolization group (*N* = 40)	*p* value	Total (*N* = 88)
Age [years, median (IQR)]	65.50 (53.75, 73.25)	59.00 (43.75, 72.50)	0.160	63.50 (52.00, 73.25)
Gender [*n* (%)]			**0.070**	
Male	33 (68.75)	34 (85.00)		67 (76.14)
Female	15 (31.25)	6 (15.00)		21 (23.86)
Concomitant diseases [*n* (%)]				
Hypertension	19 (39.58)	12 (30.00)	0.350	31 (35.23)
Diabetes mellitus	8 (16.67)	5 (12.50)	0.580	13 (14.77)
Heart failure	3 (6.25)	1 (2.50)	0.740	4 (4.55)
Liver cirrhosis	4 (8.33)	4 (10.00)	1.000	8 (9.09)
Renal failure	8 (16.67)	1 (2.50)	**0.070**	9 (10.23)
History of abdominal surgery	17 (35.42)	10 (25.00)	0.290	27 (30.68)
Alcohol [*n* (%)]	11 (22.92)	13 (32.50)	0.310	24 (27.27)
Smoking [*n* (%)]	12 (25.00)	12 (30.00)	0.600	24 (27.27)
Systolic blood pressure [mmHg, median (IQR)]	114.00 (108.00, 133.75)	111.50 (102.50, 123.00)	0.140	112.50 (103.75, 128.00)
Laboratory index				
Hemoglobin [g/L, median (IQR)]	61.50 (55.75, 75.50)	60.50 (50.75, 73.00)	0.590	61.00 (53.00, 73.50)
WBC [10^9/L, median (IQR)]	9.55 (6.43, 12.73)	8.62 (6.74, 12.95)	0.660	9.18 (6.69, 12.95)
PLT [10^9/L, median (IQR)]	162.00 (121.75, 201.25)	151.50 (115.50, 214.00)	0.670	159.00 (118.75, 206.75)
Albumin (g/L, mean ± SD)	27.53 ± 6.15	28.96 ± 5.86	0.270	28.18 ± 6.03
BUN [mmol/L, median (IQR)]	8.05 (4.90, 12.30)	7.85 (4.70, 13.50)	0.68	7.95 (4.85, 13.34)
Cr [umol/L, median (IQR)]	99.00 (61.77, 142.28)	77.25 (60.05, 91.93)	**0.070**	79.55 (61.50, 125.35)
PT [s, median (IQR)]	13.10 (12.30, 14.17)	12.85 (12.07, 14.33)	0.530	12.90 (12.20, 14.33)
INR, median (IQR)	1.12 (1.06, 1.24)	1.13 (1.04, 1.24)	0.590	1.13 (1.05, 1.24)
Endoscopy [positive found, *n* (%)]	35 (72.92)	34 (85.00)	0.170	69 (78.41)
Clip use [*n* (%)]	21 (43.75)	21 (52.50)	0.410	42 (47.73)
Time to treatment ≤6 h [*n* (%)]	30 (62.50)	23 (57.50)	0.630	53 (60.23)
Blood transfusion [*n* (%)]	36 (75.00)	36 (90.00)	**0.070**	72 (81.82)
Blood transfusion units [u, mean ± SD]	2.39 ± 2.11	4.55 ± 5.20	**0.017**	3.37 ± 3.96
Embolic materials [*n* (%)]			0.102	
Spring coil	11 (22.92)	7 (17.50)		18 (20.45)
Gelatin sponge	4 (8.33)	10 (25.00)		14 (15.91)
Spring coil and gelatin sponge	33 (68.75)	23 (57.50)		56 (63.64)

WBC: White blood cell; PLT: Platelets; BUN: Blood urea nitrogen; Cr: Creatinine; PT: Prothrombin time; INR: International normalized ratio; SD; Standard deviation; IQR: Interquartile range. Bold values indicate statistical significance at *p*< 0.05.

### Comparison of the embolized artery between super-selective embolization group and selective embolization group

As detailed in Table 2, the most frequently embolized vessels in the super-selective embolization group among patients with NVUGIB and LGIB included the gastroduodenal artery (20/67, 29.9%), left gastric artery (10/67, 14.9%), superior mesenteric artery (10/67, 14.9%), and ileocolic artery (9/67, 13.4%). In the selective embolization group, gastroduodenal artery (40.8%, 20/49), left gastric artery (18.4%, 9/49) and right gastric artery (12.2%, 6/49) were the common embolization vessels. In addition, two or more vessels were embolized in eight patients in the super-selective embolization group and five patients in the selective embolization group.

**Table 2:** Embolized artery of TAE (contains its subtle branches).

Variables	Super-selective embolization (*N* = 67)	Selective embolism (*N* = 49)
NVUGIB		
Common embolism		
Gastroduodenal artery	20	20
Left gastric artery	10	9
Right gastric artery	1	6
LGIB		
Superior mesenteric artery	10	2^#^
Inferior mesenteric artery	1	0
Jejunal artery	8	3
Ileocolic artery	9	3
Rectal artery	0	1
Cohesive embolism (embolization of 2 or more blood vessels at the same time)	8	5

#One case was jejunal ulcer with perforation, and the other case was superior mesenteric artery-portal vein fistula with hemangiomatous changes.

### Comparison of clinical outcomes between the two groups

All patients with NVUGIB were treated with super-selective or selective embolization. All cases achieved technical success. 85.42% of the super-selective embolization group achieved clinical success, and 70.00% of the selective embolization group achieved clinical success. The rebleeding rate of super-selective embolization group was 14.58% (7/48), which was slightly lower than that of selective embolization group (30.00%, 12/40), but the difference was not statistically significant (*p* = 0.080). the rebleeding within 3 days rate of the super-selective embolization group was statistically lower than that in the selective embolization group (8.33 vs. 27.50, *p* = 0.017). The super-selective embolization group showed a non-significant reduction in the need for additional therapy after rebleeding (*p* = 0.082). In the super-selective embolization group, 3 patients with rebleeding underwent endoscopic treatment. Rebleeding patients in the selective embolization group were treated endoscopically in 2 patients, embolization in 3 patients, surgery in 2 patients. There were no significant differences in postoperative adverse events between the two groups (*p >* 0.05). The median length of hospital stay was 10 (8.00–14.00) days in the super-selective embolization group and 11 (7.75–13.25) days in the selective embolization group. The mortality was 16.67% in the super-selective embolization group and 15.00% in the selective embolization group, and the difference was not statistically significant. In terms of bleeding-related mortality, superselective embolization shows a slightly lower rate than selective embolization. However, there was no statistically significant difference between the two (6.25% vs. 7.50%, *p* = 1.000) (Table 3). A multivariable analysis was performed to determine whether super-selective embolization was an independent risk factor for rebleeding after TAE (Table 4). The analysis revealed no significant association between superselective embolization and rebleeding (odds ratio: 0.40, 95% confidence interval: 0.14–1.14, *p* = 0.085), whereas the units of blood transfused showed a significant association with rebleeding (odds ratio: 1.35, 95% confidence interval:1.10–1.67, *p* = 0.005). Additionally, Supplementary Table 2 compares the outcomes between superselective and selective embolization in patients with LGIB. No significant differences were observed in clinical success, adverse events, rebleeding, or mortality between the two groups. Notably, all 5 cases of intestinal ischemia following TAE occurred in this patient subset, with 3 in the super-selective and 2 in the selective embolization group. Among these, 4 patients underwent surgical intervention and 1was managed conservatively.

**Table 3:** Comparison of clinical outcomes between the two groups of patients with NVUGIB.

Variables	Super-selective embolization group (*N* = 48)	Selective embolization group (*N* = 40)	*P* value	Total (*N* = 88)
Technical success [*n* (%)]	48 (100.00)	40 (100.00)	1.000	88(100.00)
Clinical success [*n* (%)]	41 (85.42)	28 (70.00)	0.080	69 (78.41)
Rebleeding <3 days [*n* (%)]	4 (8.33)	11 (27.50)	**0.017**	15 (17.05)
Rebleeding [*n* (%)]	7 (14.58)	12 (30.00)	0.080	19 (21.59)
Additional therapy for rebleeding			0.082	
Endoscopy [*n* (%)]	3 (6.25)	2 (5.00)		5 (5.68)
Re-embolization [*n* (%)]	0 (0.00)	3 (7.50)		3 (3.41)
Surgical treatment [*n* (%)]	0 (0.00)	2 (5.00)		2 (2.27)
Complications				
Fever [*n* (%)]	17 (35.42)	8 (20.00)	0.110	25 (28.41)
Abdominal pain [*n* (%)]	14 (29.17)	8 (20.00)	0.323	22 (25.00)
Nausea and vomiting [*n* (%)]	6 (12.50)	5 (12.50)	1.000	11 (12.50)
Hospitalization median [day, median (IQR)]	10.00 (8.00, 14.00)	11.00 (7.75, 13.25)	0.591	11.00 (8.00, 14.00)
Mortality, [*n* (%)]	8 (16.67)	6 (15.00)	0.831	14 (15.91)
Bleeding ralated mortality, *n*(%)	3 (6.25)	3 (7.50)	1.000	6 (6.82)

IQR: interquartile range. Bold values indicate statistical significance at *p*< 0.05.

**Table 4:** Univariable and multivariable analyses examining rebleeding as the primary outcome in patients with NVUGIB.

Variables	Univariable OR (95% CI)	*p* value	Multivariable OR (95% CI)	*p* value
Super-selective embolization	0.40 (0.14 ~ 1.14)	0.085		
Age	1.00 (0.97 ~ 1.04)	0.803		
Gender (Female)	0.82 (0.24 ~ 2.79)	0.653		
Alcohol intake, yes	1.31 (0.43 ~ 3.95)	0.635		
Cigarette smoking, yes	0.65 (0.19 ~ 2.21)	0.494		
Diabetes mellitus	1.11 (0.27 ~ 4.50)	0.888		
Hypertension	1.45 (0.51 ~ 4.11)	0.480		
Hemoglobin	0.98 (0.94 ~ 1.01)	0.139		
WBC	1.00 (0.88 ~ 1.13)	0.954		
PLT	1.00 (0.99 ~ 1.00)	0.270		
Albumin	0.93 (0.84 ~ 1.02)	0.110		
BUN	1.03 (0.96 ~ 1.10)	0.450		
Cr	1.00 (1.00 ~ 1.01)	0.249		
PT	1.12 (1.00 ~ 1.26)	0.051		
INR	3.49 (0.99 ~ 12.26)	0.051		
Blood transfusion units	1.35 (1.10 ~ 1.67)	**0.005**	1.35 (1.10 ~ 1.67)	**0.005**
Time to treatment ≤6 h	2.15 (0.70 ~ 6.64)	0.182		
Embolic materials				
Spring coil	1.00 (Reference)	-		
Gelatin sponge	1.36 (0.23 ~ 8.08)	0.733		
Spring coil and gelatin sponge	1.51 (0.38 ~ 6.05)	0.559		
Single embolization	0.80 (0.19 ~ 3.30)	0.758		
Endoscopy	1.04 (0.30 ~ 3.61)	0.949		
Clip use	0.98 (0.36 ~ 2.71)	0.972		

WBC: White blood cell; PLT: Platelets; BUN: Blood urea nitrogen; Cr: Creatinine; PT: Prothrombin time; INR: International normalized ratio. Bold values indicate statistical significance at *p*< 0.05.

### Comparison of clinical outcomes among patients receiving different embolic materials during TAE

As summarized in Table 5, clinical outcomes were compared among patients receiving spring coils, gelatin sponge, or a combination of both during TAE for NVUGIB. No significant differences were observed across any outcome measure, including technical success (100% in all groups), clinical success (78.41% overall), rebleeding, complication rates, or mortality (all *p* > 0.05). In contrast, among patients with LGIB (Supplementary Table 3), the embolic material showed a significant impact on early rebleeding (*p* = 0.041), with the highest rate observed in the combination therapy group (45.45%). Consequently, the need for additional therapy for rebleeding also differed significantly among the groups (*p* = 0.027).

**Table 5:** Clinical outcomes by embolic material used in TAE in patients with NVUGIB.

Variables	Spring coil (*N* = 18)	Gelatin sponge (*N* = 14)	Spring coil and gelatin sponge (*N* = 56)	*P* value	Total (*N* = 88)
Technical success [*n* (%)]	18 (100.00)	14 (100.00)	56 (100.00)	1.000	88 (100.00)
Clinical success [*n* (%)]	15 (83.33)	11 (78.57)	43 (76.79)	0.930	69 (78.41)
Rebleeding <3 days [*n* (%)]	2 (11.11)	3 (21.43)	10 (17.86)	0.714	15 (17.05)
Rebleeding [*n* (%)]	3 (16.67)	3 (21.43)	13 (23.21)	0.930	19 (21.59)
Additional therapy for rebleeding				0.919	
Endoscopy [*n* (%)]	1 (5.56)	0 (0.00)	4 (7.14)		5 (5.68)
Re-embolization [*n* (%)]	0 (0.00)	1 (7.14)	2 (3.57)		3 (3.41)
Surgical treatment [*n* (%)]	0 (0.00)	0 (0.00)	2 (3.57)		2 (2.27)
Complications					
Fever [*n* (%)]	3 (16.67)	3 (21.43)	19 (33.93)	0.302	25 (28.41)
Abdominal pain [*n* (%)]	4 (22.22)	1 (7.14)	17 (30.36)	0.179	22 (25.00)
Nausea and vomiting [*n* (%)]	3 (16.67)	2 (14.29)	6 (10.71)	0.721	11 (12.50)
Hospitalization median [day, median (IQR)]	11.50 (8.50,15.75)	11.50 (7.25,16.50)	9.00 (8.00,13.00)	0.391	11.00 (8.00, 14.00)
Mortality, [*n* (%)]	3 (16.67)	1 (7.14)	10 (17.86)	0.704	14 (15.91)
Bleeding ralated mortality, *n*(%)	1 (5.56)	1 (7.14)	4 (7.14)	0.339	6 (6.82)

IQR: Interquartile range.

## Discussion

NVGIB is one of the common acute and critical conditions in clinical practice, and it’s incidence rate has gradually decreased in recent years (18). Endoscopic hemostasis is a common and effective hemostasis method, but its rebleeding rate is still very high, approximately 5–15% of patients cannot stop bleeding through endoscopy and require TAE or surgery (18, 19). TAE is a very effective intervention measure for managing NVGIB. Compared with surgical operations, it provides a more minimally invasive alternative for bleeding failed hemostasis under endoscopy. Although the rebleeding rate of TAE is higher compared with surgery, the risk of complications of TAE is significantly lower and the length of hospital stay is shorter (4). In a British national audit, the mortality rate of salvage surgery after NVGIB endoscopic hemostasis failure remained as high as 23%, while that of TAE was 10% (20). Therefore, TAE is the preferred salvage therapy for patients who have failed endoscopic treatment.

Due to concerns that the embolized intestinal segment may be infarcted, there has always been a debate on the most suitable embolizing agent, location and device for TAE, among which the selectivity of the embolizing location is very important (21, 22). It is crucial to select the most selective embolization location as much as possible to minimize the risk of ischemia. The safety and efficacy of super-selective embolization in the treatment of acute lower gastrointestinal bleeding have been reported by several studies, while there are still relatively few studies on acute upper gastrointestinal bleeding (21, 23–27).

This study focuses on the outcomes of TAE in patients with NVGIB. We found that super-selective embolization of NVUGIB resulted in a significantly lower rate of early rebleeding (within 3 days) than selective embolization, which was similar to the result of a previous study (24). Importantly, the average blood transfusion unit was identified as an independent predictor of rebleeding, consistent with previous literature showing that higher transfusion volume was associated with clinical failure in TAE. Previous studies have shown that the use of higher units of red blood cell concentrate is associated with clinical failure of TAE (28). Although the overall clinical success rate was higher in the super-selective group, this difference did not reach statistical significance, possibly due to the sample size. However, no outcome difference was observed between the two groups in LGIB.

In the choice of embolic agent, we usually use gelatin sponge for prophylactic embolization in patients with unclear bleeding site, and spring coil or spring coil combined with gelatin sponge for patients with clear bleeding site. This selection bias might lead to an overestimation of the clinical efficacy of gelatin sponge when used alone. In UGIB subgroup, the type of embolic agent did not significantly influence clinical success, rebleeding, or complication rates. In contrast, within the LGIB subgroup, the choice of embolic material significantly affected the rate of early rebleeding and the subsequent need for additional therapy. As in previous studies, we found that the combination of spring coil and gelatin sponge was superior to spring coil alone (15).

Due to the insufficient collateral blood supply of the lower gastrointestinal blood vessels, the ischemia rate after embolization of lower gastrointestinal bleeding is higher than that after embolization of the upper gastrointestinal tract (29). In this study, intestinal ischemia occurred after embolization treatment in patients with lower gastrointestinal bleeding. In a study on the treatment of acute small intestinal bleeding, it was found that super-selective embolism was an important prognostic factor associated with fewer major complications (30). In a study of TAE in the treatment of acute lower gastrointestinal bleeding, it was found that super-selective embolization is an important prognostic factor associated with reduced recurrent bleeding and fewer major complications (24). However, in this study, it was not observed that the postoperative intestinal ischemia rate in the super-selective embolization group was significantly lower than that in the selective embolization group. This might be because more cases of lower gastrointestinal bleeding in this study chose super-selective embolization. There were no significant differences in the incidence of postoperative adverse events, hospital stay and mortality between the two groups of patients.

This study has several limitations. Firstly, its retrospective nature introduces potential selection bias. Secondly, the sample size was small, particularly in the LGIB subgroup, may have been underpowered to detect significant differences in some outcomes, such as the rate of intestinal ischemia. Thirdly, embolic materials were not randomized, and their influence, especially in LGIB, warrants further investigation. Finally, the diagnosis of intestinal ischemia was only confirmed in clinically suspected cases via CT or surgery, potentially underestimating its true incidence.

## Conclusion

In conclusion, for the management of refractory NVUGIB, super-selective TAE is associated with a significant reduction in early rebleeding compared to selective TAE, without increasing the risk of mortality. The embolization technique itself was not an independent predictor of rebleeding, whereas a higher transfusion requirement was. When embolizing for LGIB, clinicians must remain vigilant about the risk of intestinal ischemia. The choice of embolic agent appears to have a more pronounced effect on outcomes in LGIB than in UGIB.

## Data Availability

The raw data supporting the conclusions of this article will be made available by the authors, without undue reservation.
