# Supplementary material for: Comparison of the efficacy and safety of super-selective and selective transcatheter arterial embolization in non-variceal gastrointestinal bleeding
Source: Front Med (Lausanne). 2025 Dec 1;12:1697511. doi: 10.3389/fmed.2025.1697511 (PMC12702910; doi:10.3389/fmed.2025.1697511)
Supplement: Supplementary file 1 [file Data_Sheet_1.docx]

| **Supplementary Table 1 .Baseline characteristics of patients with LGIB** | | | | |
| --- | --- | --- | --- | --- |
| **Variables** | **Super-selective embolization group (N=19)** | **Selective embolization group(N=9)** | ***P* value** | **Total (N =28)** |
| **Age (years, median (IQR))** | 66.00 (53.50, 69.00) | 50.00 (43.00, 63.00) | 0.058 | 63.00 (49.00, 68.25) |
| **Gender [n (%)]** |  |  | 1.000 |  |
| Male | 12 (63.16) | 6 (66.67) |  | 18 (64.29) |
| Female | 7 (36.84) | 3 (33.33) |  | 10 (35.71) |
| **Concomitant diseases [n (%)]** |  |  |  |  |
| Hypertension | 7 (36.84) | 2 (22.22) | 0.670 | 9 (32.14) |
| Diabetes mellitus | 1 (5.26) | 2 (22.22) | 0.234 | 3 (10.71) |
| Heart failure | 1 (5.26) | 0 (0.00) | 1.000 | 1 (3.57) |
| Liver cirrhosis | 1 (5.26) | 2 (22.22) | 0.234 | 3 (10.71) |
| Renal failure | 2 (10.53) | 0 (0.00) | 1.000 | 2 (7.14) |
| History of abdominal surgery | 2 (10.53) | 1 (11.11) | 1.000 | 3 (10.71) |
| Alcohol [n (%)] | 4 (21.05) | 1 (11.11) | 1.000 | 5 (17.86) |
| Smoking [n (%)] | 3 (15.79) | 3 (33.33) | 0.352 | 6 (21.43) |
| Systolic blood pressure (mmHg, median (IQR)) | 122.00 (103.50, 131.00) | 102.00 (99.00, 142.00) | 0.922 | 118.50 (102.00, 136.25) |
| **Laboratory index** |  |  |  |  |
| Hemoglobin (g/L, median (IQR)) | 64.00 (53.00, 69.00) | 65.00 (55.00, 75.00) | 0.506 | 64.00 (53.00, 71.50) |
| WBC (10^9/L, median (IQR)) | 8.10 (6.12, 10.14) | 9.98 (5.04, 11.50) | 0.438 | 8.52 (5.65, 10.65) |
| PLT (10^9/L,median (IQR)) | 121.00 (95.50, 202.50) | 168.00 (125.00, 251.00) | 0.205 | 136.50 (99.75, 211.25) |
| Albumin (g/L, mean ± SD) | 26.66 ± 5.84 | 28.01 ± 8.26 | 0.621 | 27.09 ± 6.58 |
| BUN [mmol/L,median (IQR)] | 5.60 (3.95, 7.50) | 4.20 (4.00, 5.60) | 0.201 | 4.95 (3.95, 7.32) |
| Cr [umol/L, median (IQR)] | 82.20 (59.60, 109.20) | 64.20 (48.00, 69.20) | 0.110 | 68.20 (52.68, 92.80) |
| PT [s, median (IQR)] | 13.10 (12.40, 15.15) | 14.80 (13.80, 18.30) | 0.121 | 13.95 (12.60, 15.53) |
| INR, median (IQR) | 1.12 (1.04, 1.35) | 1.25 (1.08, 1.29) | 0.676 | 1.18 (1.05, 1.30) |
| **Endoscopy [Positive found, n (%)]** | 5 (26.32) | 3 (33.33) | 1.000 | 8 (28.57) |
| **Clip use [n (%)]** | 0 (0.00) | 2 (22.22) | 0.095 | 2 (7.14) |
| **Time to treatment ≤6h [n (%)]** | 12 (63.16) | 9 (100.00) | 0.062 | 21 (75.00) |
| **Blood transfusion [n (%)]** | 16 (84.21) | 6 (66.67) | 0.352 | 22 (78.57) |
| **Blood transfusion units [u, mean ± SD]** | 2.00 (1.75, 4.50) | 2.00 (0.00, 7.00) | 0.880 | 2.00 (1.50, 5.00) |
| **Embolic materials [n (%)]** |  |  | 0.351 |  |
| Spring coil | 6 (31.58) | 1 (11.11) |  | 7 (25.00) |
| Gelatin sponge | 5 (26.32) | 5 (55.56) |  | 10 (35.71) |
| Spring coil and gelatin sponge | 8 (42.11) | 3 (33.33) |  | 11 (39.29) |

**Abbreviations:**WBC: White blood cell; PLT: Platelets; BUN: Blood urea nitrogen; Cr: Creatinine; PT: Prothrombin time; INR: International normalized ratio; SD; Standard deviation; IQR: Interquartile range.

| **Supplementary Table 2. Comparison of clinical outcomes between the two groups of patients with LGIB** | | | | |
| --- | --- | --- | --- | --- |
| **Variables** | **Super-selective embolization group (N=19)** | **Selective embolization group (N=9)** | ***P* value** | **Total (N =28)** |
| **Technical success [n (%)]** | 19 (100.00) | 9 (100.00) | 1.000 | 28(100.00) |
| **Clinical success [n (%)]** | 13 (68.42) | 6 (66.67) | 1.000 | 19 (67.86) |
| **Rebleeding <3 days [n (%)]** | 3 (15.79) | 2 (22.22) | 1.000 | 5 (17.86) |
| **Rebleeding [n (%)]** | 5 (26.32) | 3 (33.33) | 1.000 | 8 (28.57) |
| **Additional therapy for rebleeding** |  |  | 0.363 |  |
| Endoscopy [n (%)] | 2 (10.53) | 0 (0.00) |  | 2 (7.14) |
| Re-embolization [n (%)] | 0 (0.00) | 1 (11.11) |  | 1 (3.57) |
| Surgical treatment [n (%)] | 2 (10.53) | 2 (22.22) |  | 4 (14.29) |
| **Complications** |  |  |  |  |
| Fever [n (%)] | 5 (26.32) | 2 (22.22) | 1.000 | 7 (25.00) |
| Abdominal pain [n (%)] | 9 (47.37) | 2 (22.22) | 0.249 | 11 (39.29) |
| Nausea and vomiting [n (%)] | 3 (15.79) | 2 (22.22) | 1.000 | 5 (17.86) |
| Intestinal ischemia [n (%)] | 3 (15.79) | 2 (22.22) | 1.000 | 5 (17.86) |
| **Hospitalization median [day, median (IQR)]** | 10.00 (8.00, 16.50) | 12.00 (11.00, 12.00) | 0.430 | 11.00 (8.00, 14.75) |
| **Mortality, [n (%)]** | 4 (21.05) | 0 (0.00) | 0.273 | 4 (14.29) |
| **Bleeding Ralated Mortality, n(%)** | 1 (5.26) | 0 (0.00) | 1.000 | 1 (3.57) |

**Abbreviations:**IQR: Interquartile range.

| **Supplementary Table 3. Clinical Outcomes by Embolic Material Used in TAE in patients with LGIB** | | | | | |
| --- | --- | --- | --- | --- | --- |
| **Variables** | **Spring coil**  **(N=7)** | **Gelatin sponge**  **(N=10)** | **Spring coil and gelatin sponge**  **(N=11)** | ***P* value** | **Total (N =28)** |
| **Technical success [n (%)]** | 7 (100.00) | 10(100.00) | 11(100.00) | 1.000 | 28(100.00) |
| **Clinical success [n (%)]** | 5 (71.43) | 9 (90.00) | 5 (45.45) | 0.134 | 19 (67.86) |
| **Rebleeding <3 days [n (%)]** | 2 (28.57) | 0 (0.00) | 5 (45.45) | **0.041** | 7 (25.00) |
| **Rebleeding [n (%)]** | 2 (28.57) | 1 (10.00) | 5 (45.45) | 0.279 | 8 (28.57) |
| **Additional therapy for rebleeding** |  |  |  | **0.027** |  |
| Endoscopy [n (%)] | 2 (28.57) | 0 (0.00) | 0 (0.00) |  | 2 (7.14) |
| Re-embolization [n (%)] | 0 (0.00) | 0 (0.00) | 1 (9.09) |  | 1 (3.57) |
| Surgical treatment [n (%)] | 2 (28.57) | 0 (0.00) | 2 (18.18) |  | 4 (14.29) |
| **Complications** |  |  |  |  |  |
| Fever [n (%)] | 1 (14.29) | 2 (20.00) | 4 (36.36) | 0.634 | 7 (25.00) |
| Abdominal pain [n (%)] | 4 (57.14) | 5 (50.00) | 2 (18.18) | 0.206 | 11 (39.29) |
| Nausea and vomiting [n (%)] | 1 (14.29) | 2 (20.00) | 2 (18.18) | 1.000 | 5 (17.86) |
| **Hospitalization median [day, median (IQR)]** | 13.00 (8.00,20.50) | 10.00 (8.25,12.00) | 11.00 (9.50,16.50) | 0.753 | 11.00 (8.00, 14.75) |
| **Mortality, [n (%)]** | 0 (0.00) | 2 (20.00) | 2 (18.18) | 0.643 | 4 (14.29) |
| **Bleeding Ralated Mortality, n(%)** | 0 (0.00) | 0 (0.00) | 1 (9.09) | 1.000 | 1 (3.57) |

**Abbreviations:**IQR: Interquartile range.
